# Supplementary material for: Learning curve and surgical outcome of robotic assisted colorectal surgery with ERAS program
Source: Sci Rep. 2022 Nov 29;12:20566. doi: 10.1038/s41598-022-24665-w (PMC9709162; doi:10.1038/s41598-022-24665-w)
Supplement: Supplementary file 2 — Supplementary Information 2. [file 41598_2022_24665_MOESM2_ESM.zip › RAL Raw data-Ñ[▒K/090/OP N_ñw╝╨░O▒Kñσ.pdf]

W76 / W76

臺中榮民總醫院
手術室護理記錄

[redacted]

基本資料

- 室別： 開刀房-17室，手術科別： CRS，手術類別： 常規，日期： 2021/06/05
- 進入時間： 0800 ； 麻醉開始時間： 0800 ； Time Out時間： 0915 ； 劃刀時間： 0917 ； 手術結束時間： 1230 送出時間： 1305 ；
- 麻醉方式： GE

  診斷：Malignant tumor of sigmoid colon 25CM FAV

  手術名稱：Robot Assisted Colectomy(L)

  手術局部用藥：NIL

  術前預防性抗生素：
  - 抗生素名稱一： Veterin 1GM IVD由麻醉護理師執行
  - 時間： 0900
  - 上次簽章: 李芩瑜

  ，追加預防性抗生素：
- 手術醫師： 蔣鋒帆，楊曙榮，陳瑢夫
- 麻醉醫師： VS 沈靜慧
-
  - 刷手護士A ： 李郁萱，時間： 0800，刷手護士B ： 陳慧瑜，時間： 0830，接替刷手一： 李芩瑜，時間： 1130
  - 流動護士A ： 李芩瑜，時間： 0800，接替流動一： 陳慧瑜，時間： 1100-1200，接替流動二： 李郁萱，時間： 1200

皮膚狀況

A-V Shunt： 無

術前： 完整

記錄者： 上次簽章: 李芩瑜

術後： 完整

記錄者： 上次簽章: 李郁萱

手術準備

手術臥式 單一手術臥式

-
  - 手術臥式： 截石臥式
  - 使用附件： 溫毯 38 ℃，頭圈，腳架，其它： 海綿墊、Latter Suport
  - 皮膚準備： Hibiscrub，2% Chlorhexidine
  - 電燒： 使用電燒: 電極片位置： 右大腿，雙極電燒，記錄者： 上次簽章: 李芩瑜

止血帶T： 無

植入物

- 植入物名稱/號碼/數量/部位：Da Vinci ENDO GIA:45mm*1/腸斷處
  EEA:29mm*1/腸吻合處
  Surgiwrap可吸收防沾黏:00.2x100x130mm*1/腹腔

，記錄者： 上次簽章: 陳慧瑜

標本送檢

- 細菌培養 無
- 細胞學 無
- 冰凍切片 無
- 病理標本

  組織名稱一：S colon

  ，

  組織名稱二：IMA root

  ，

組織名稱三：distal cut end

- 截肢 無

標本交付 標本： 3 袋 ，標本： 0 盒 ，標本： 0 瓶 ，細胞學： 0 管 ，Culture Tube： 0 管 ，被交付醫師： R 陳璿夫，

- 記錄者： 上次簽章: 李郁萱
- 時間： 1225

傷口術後敷料

傷口敷料： 傷口敷料： 無

- 請於圖上標示部位：

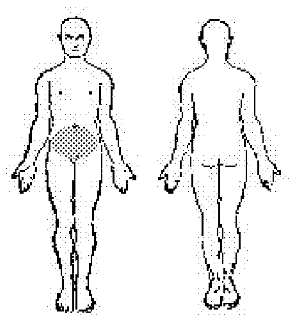

Trocar 洞

- 傷口敷料： 紗布、Mpore

導管

導管	天數	導管資訊	插入日期	插入時間	更換日期	拔除日期	拔除時間	拔除原因	預計拔除/更換	預計拔除/更換日期
Epidural PCA(1)	1	部位:硬脊膜;	2021/06/05	0820						
Foley(1)	1	號碼:16 Fr.; 部位:膀胱;	2021/06/05	0840						
Double Lumen CVC(1)	1	號碼:8 Fr.; 深度:15cm; 部位:jugular (右); 象限:RU;	2021/06/05	0835						
A-line (1)	1	號碼:20 G; 部位:左手;	2021/06/05	0830						

用物計數

- 手術中出血量： Minimal c.c.
- 手術中尿量： 1100 c.c.
-
    - 其它： 無

用物計數： 多切口同時縫合

- 1.器械： 正確，2.縫針： 正確，3.刀片： 正確，4.紗布： 正確，5.腹部墊： 未使用，6.紗球： 未使用，7.PEANUTS ： 未使用，9.絲帶： 未使用，10.針頭： 未使用，11.Vessel loop： 未使用，12.Boots： 未使用，13.Jelco： 未使用，14.棉條： 未使用，
    - 第一次：
        - 刷手護士 ： 李苓瑜
        - 流動護士： 上次簽章: 陳慧瑜
        - 時間： 1135

    ,
    - 第二次：
        - 刷手護士 ： 李苓瑜
        - 流動護士： 上次簽章: 陳慧瑜
        - 時間： 1150

    ,
    - 第三次：
        - 刷手護士 ： 李苓瑜
        - 流動護士： 上次簽章: 李郁萱

- 時間： 1225

備註

備註： DAVINCI 30度 SCOPE NO.1

- 記錄者： 上次簽章: 李郁萱
- 時間： 1305

填寫護理人員 李郁萱

正式摘要

W76 / W76

doctorRank:B
